# Unveiling public perceptions at the beginning of lockdown: an application of structural topic modeling and sentiment analysis in the UK and India

**DOI:** 10.1186/s12889-024-20160-1

**Published:** 2024-10-15

**Authors:** Xinhe Kang, Panagiotis Stamolampros

**Affiliations:** School of Business, Hebei University of Engineering Science, Shijiazhuang, Hebei, People’s Republic of China

**Keywords:** COVID-19, Lockdown, Social media, Lexicon-based sentiment analysis, Structural topic modeling

## Abstract

**Background:**

The appearance of the COVID-19 virus in December 2019, quickly escalated into a global crisis, prompting the World Health Organization to recommend regional lockdowns. While effective in curbing the virus’s spread, these measures have triggered intense debates on social media platforms, exposing widespread public anxiety and skepticism. The spread of fake news further fueled public unrest and negative emotions, potentially undermining the effectiveness of anti-COVID-19 policies. Exploring the narratives surrounding COVID-19 on social media immediately following the lockdown announcements presents an intriguing research avenue. The purpose of this study is to examine social media discourse to identify the topics discussed and, more importantly, to analyze differences in the focus and emotions expressed by the public in two countries (the UK and India). This is done with an analysis of a big corpus of tweets.

**Methods:**

The datasets comprised of COVID-19-related tweets in English, published between March 29th and April 11th 2020 from residents in the UK and India. Methods employed in the analysis include identification of latent topics and themes, assessment of the popularity of tweets on topic distributions, examination of the overall sentiment, and investigation of sentiment in specific topics and themes.

**Results:**

Safety measures, government responses and cooperative supports are common themes in the UK and India. Personal experiences and cooperations are top discussion for both countries. The impact on specific groups is given the least emphasis in the UK, whereas India places the least focus on discussions related to social media and news reports. Supports, discussion about the UK PM Boris Johnson and appreciation are strong topics among British popular tweets, whereas confirmed cases are discussed most among Indian popular tweets. Unpopular tweets in both countries pay the most attention to issues regarding lockdown. According to overall sentiment, positive attitudes are dominated in the UK whilst the sentiment is more neutral in India. Trust and anticipation are the most prevalent emotions in both countries. In particular, the British population felt positive about community support and volunteering, personal experiences, and government responses, while Indian people felt positive about cooperation, government responses, and coping strategies. Public health situations raise negative sentiment both in the UK and India.

**Conclusions:**

The study emphasizes the role of cultural values in crisis communication and public health policy. Individualistic societies prioritize personal freedom, requiring a balance between individual liberty and public health measures. Collectivistic societies focus on community impact, suggesting policies that could utilize community networks for public health compliance. Social media shapes public discourse during pandemics, with popular and unpopular tweets reflecting and reshaping discussions. The presence of fake news may distort topics of high public interest, necessitating authenticity confirmation by official bloggers. Understanding public concerns and popular content on social media can help authorities tailor crisis communication to improve public engagement and health measure compliance.

## Background

### Introduction

The COVID-19 pandemic started in December 2019 [1]. The Chinese New Year holiday, which saw people flocked back to their hometowns, fueled the virus’ spread across China. To curb this grave situation, traffic routes in Wuhan and most of the provinces were closed by the end of January 2020, leading to the announcement of a nationwide lockdown in early February. However, the virus couldn’t be contained within China’s borders and soon was spread worldwide, resulting in a pandemic with a plethora of confirmed cases and reported deaths. To curb the spread of the virus, the World Health Organization (WHO) recommended lockdowns and mandatory mask-wearing as preventive measures. Lockdowns had several consequences, including isolation and the cancellation of public events.

Several studies have demonstrated the effectiveness of lockdown measures by revealing a significant decline in the growth rate of confirmed cases, thus mitigating the spread of the pandemic [2–5]. Studies, e.g [6, 7], find that many countries have benefited from this preventive policy. However, throughout the period of quarantine, this measure has sparkled heated online discussions on social media platforms, depicting the prevailing anxiety experienced among the public, while causing mistrust towards lockdown measures and other interventions [8]. With the exponentially increasing number of relevant tweets and other social media posts, fake news was widely spread online, generating unnecessary unrest among the public [8] and triggering people’s negative emotions [9]. High levels of public anxiety could potentially undermine the effectiveness of anti-COVID-19 policies, as some people were less willing to comply with them [10].

Social media can serve as a valuable data source for identifying and gaining insights into public discourse about the pandemic during Covid-19, thus aiding in crisis communication and health promotion messaging [11]. Undeniably, popular tweets, defined as those that receive more reaction and shared by a large number of users, had a significant influence in public perception regarding Covid-19. For instance, popular English tweets exhibited increased polarization in discussions, featuring a mix of positive and negative sentiments towards vaccines, cognitively affecting a part of the population and thus hindering the vaccination campaign [12]. Less popular tweets might not attain broad visibility, yet they can still offer valuable insights into public sentiments. attitudes to government policies [13] and evolvement of people’s sentiment [14] during the lockdown.

To date, extant research has examined social media narratives to unravel people’s concerns [15–17], to measure people’s feelings of panic [18, 19] and to analyze the role of social media in their perceptions on the Covid-19 [20, 21]. However, there is a lack of studies that shed light to the tweets published during lockdown implementation. Interestingly, some topics in above-mentioned studies have been found to be sentimental [e.g., 20, 21], while people’s emotions on other topics remain unclear. Moreover, the existing research provides a static snapshot of prevalent themes, but a comprehensive investigation into how these topics have evolved, gained prominence, or diminished throughout different phases of the pandemic would offer a clearer understanding. Another limitation of current studies in the area is that they neglect variations in topic discussion as a result of individual characteristics, such as age, socio-economic status, and cultural influences.

To fill the research gaps, our study aims to shed light on public discussions and sentiment towards COVID-19 at the start of lockdown implementation in the UK and India. By utilizing structural topic modeling and lexicon-based sentiment analysis, the study first extracts hidden topics and analyzes them in relation to their popularity, and subsequently identifies the overall sentiment and emotion types. We further categorize the sentiment expressed in each specific topic. In particular, the study answers the following research questions:

- RQ1. What is the difference in the topics on Covid-19 between the UK and India at the start of lockdown?
- RQ2. What is the difference in the topics on Covid-19 between popular and unpopular tweets at the start of lockdown?
- RQ3. What is the difference in people’s attitudes toward COVID-19 between the UK and India at the start of lockdown?

This study focuses on two countries, the United Kingdom and India. The lockdown measures were announced almost simultaneously in both countries (Mar. 23rd in the UK and 24th in India). A striking difference is that a similar timing of lockdown announcement brings distinct results, where lockdown is considered effective intervention in socioeconomically advantaged countries such as the UK [22]. Another reason for the selection of these two countries is that the population of those countries had a high number of tweets on this topic [23], from which the UK and India display different patterns of emotion distribution surrounding lockdown announcement, with more negative attitudes present in India [24].

The findings of the study are anticipated to offer a deeper understanding of people’s key concerns immediately after the lockdown announcement, assisting decision-makers in making informed choices. By analyzing social media data, this study provides a comprehensive overview of the public’s attitudes and responses to government intervention and enhances the clarity of text data to support decision-making.

## Methods

### Data

To answer our research questions, textual data was collected from the dataset on Kaggle [25] using several hashtags commonly related to COVID-19: *#coronavirus*,* #coronavirusoutbreak*,* #coronavirusPandemic*,* #covid19*,* #covid_19*,* #epitwitter* and *#ihavecorona*. We retained English tweets published between Mar. 29th and Apr. 11th, the following two weeks right after the implementation of lockdown in both countries, to investigate initial public responses. In line with the criterion in [26], popular tweets were defined as those with at least one retweet during the period. After removing duplicated tweets, we prepared 27,331 (48.12%) tweets from the UK and 29,484 (51.88%) tweets from India for further analysis. Table 1 displayed a complete structure of the data, showing that around one third of the total tweets were regarded as popular ones in both countries.

**Table 1:** Structure of the data

Countries	Total	Popular	Unpopular
The UK	27,331	9,454 (34.59%)	17,877 (65.41%)
India	29,484	9,776 (33.16%)	19,708 (66.84%)

### Methods

Topic models, based on the Bayesian analysis, allow researchers to uncover latent topics among a collection of texts. Although latent Dirichlet al.location, or LDA [27], is currently one of the most widely adopted topic models, its limitation lies in the disability to assess the effect of additional data on the texts that it complements to (i.e., the popularity of tweets in this case). To identify hidden topics among both popular and individual tweets, we applied structural topic modeling, or STM [28]. The major difference between STM and LDA lies in the addition of document metadata in STM. This metadata refers to the covariates of raw textual data and can provide additional information. The integration of textual data and metadata is beneficial for identifying topics in a document and for analyzing the relationships between variables that may impact the text written by users.

**Fig. 1:**
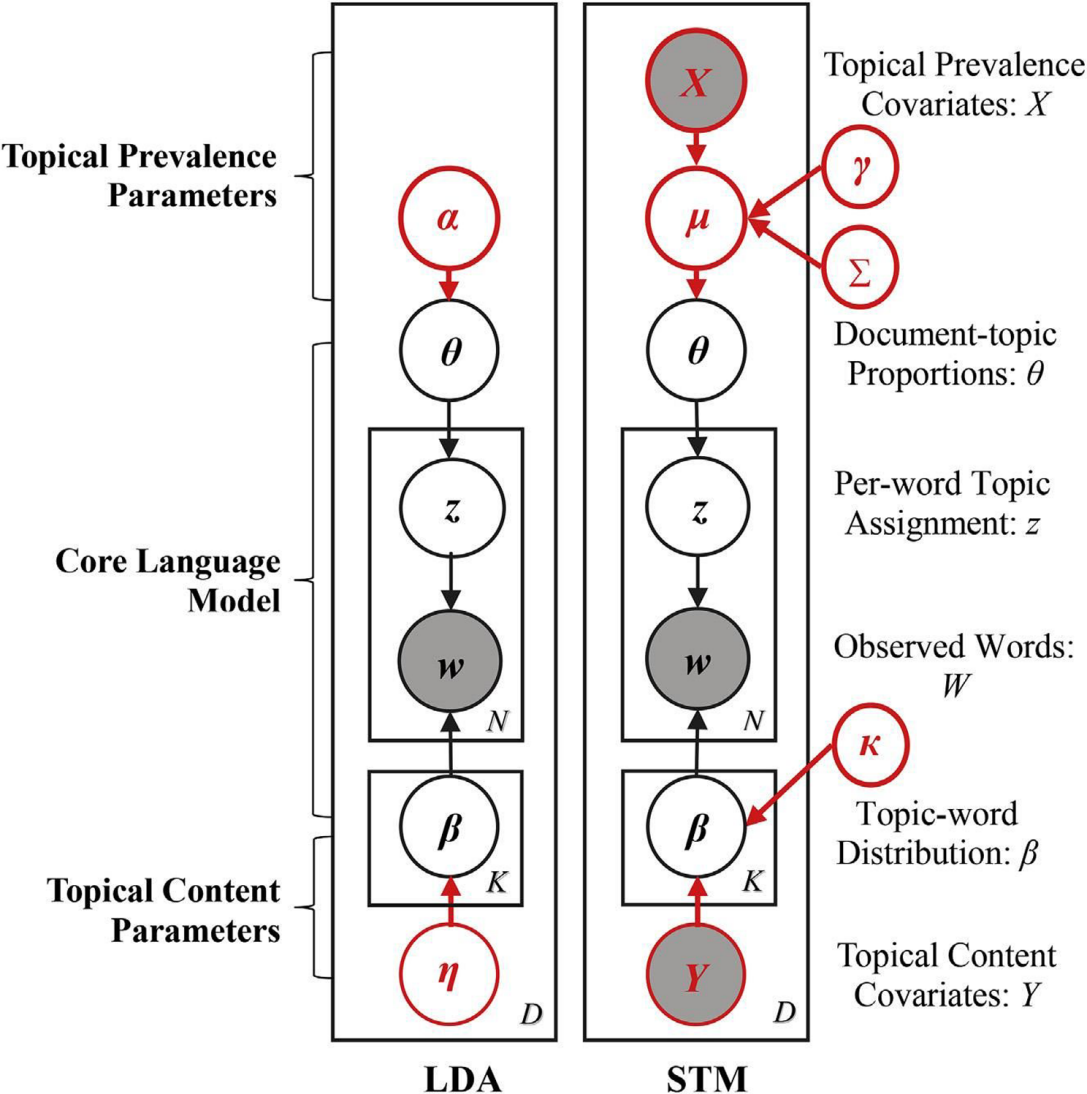
Comparison between LDA and STM [29] (p.420)

STM addresses the limitations of LDA by allowing control of both the distribution of topics in a document and the distribution of terms within topics with the use of a simple Dirichlet prefix. This results in a deeper understanding of the impact of covariates on textual data, the distribution of topics in a document, and the distribution of terms within a specific topic. Figure 1 highlights the difference in workflow. The topic distribution is not determined by the Dirichlet prior (α) but is affected by one or more document-level covariates (X), so the covariates (X) follow the logistic normal prior, which can also be expressed as $\:[\theta\:\:\sim\:Logistic\,Normal\:(X\left)\right]$ [23].

The study also performs a lexicon-based sentiment analysis. Sentiment lexicon is based on a list of lexical features (e.g., words) which are labeled according to their semantic orientation as either positive, negative, or neutral [29–31], based on which the sentiment of a text is calculated. By providing a fine-grained investigation, lexicon-based sentiment analysis has been proved to be an effective method to examine public concerns and monitor people’s mental health during the outbreak of pandemics [e.g., 32–34].

#### Data preprocessing

The corpus was pre-processed following previous studies [35, 36]. The pre-processing steps were automatically achieved by means of the R package *#tm* [37] including data cleaning, transformation, stemming, and part-of-speech tagging. During process of data cleaning, punctuations, non-English characters and stop words were removed from the tweets. Note that stop words refers to functional words whose presence do not offer essential information although dominate the corpus. Moreover, the following stages of preprocessing converted all words to lowercase letters for better recognition, stemmed words back to their root forms to reduce the number of dimensions, and tagged part-of-speech onto the words to improve the accuracy of analysis.

#### Identification of latent topics and assessment of the effect of popularity

This study aimed to explore the topics preferred by the public in the UK and India, and further examine the differences in the topics between popular and unpopular tweets by combining each tweet with its corresponding number of retweets as metadata. To achieve this, we used the R package *#stm* [28]. This package represents the corpus in three parts: a document list containing word indices and their associated counts, a vocabulary character vector containing the words associated with the word indices, and a metadata matrix containing document covariates. The package defined a data generating process for each document and then used the data to find the most likely values for the parameters within the model with the inclusion of metadata.

**Fig. 2:**
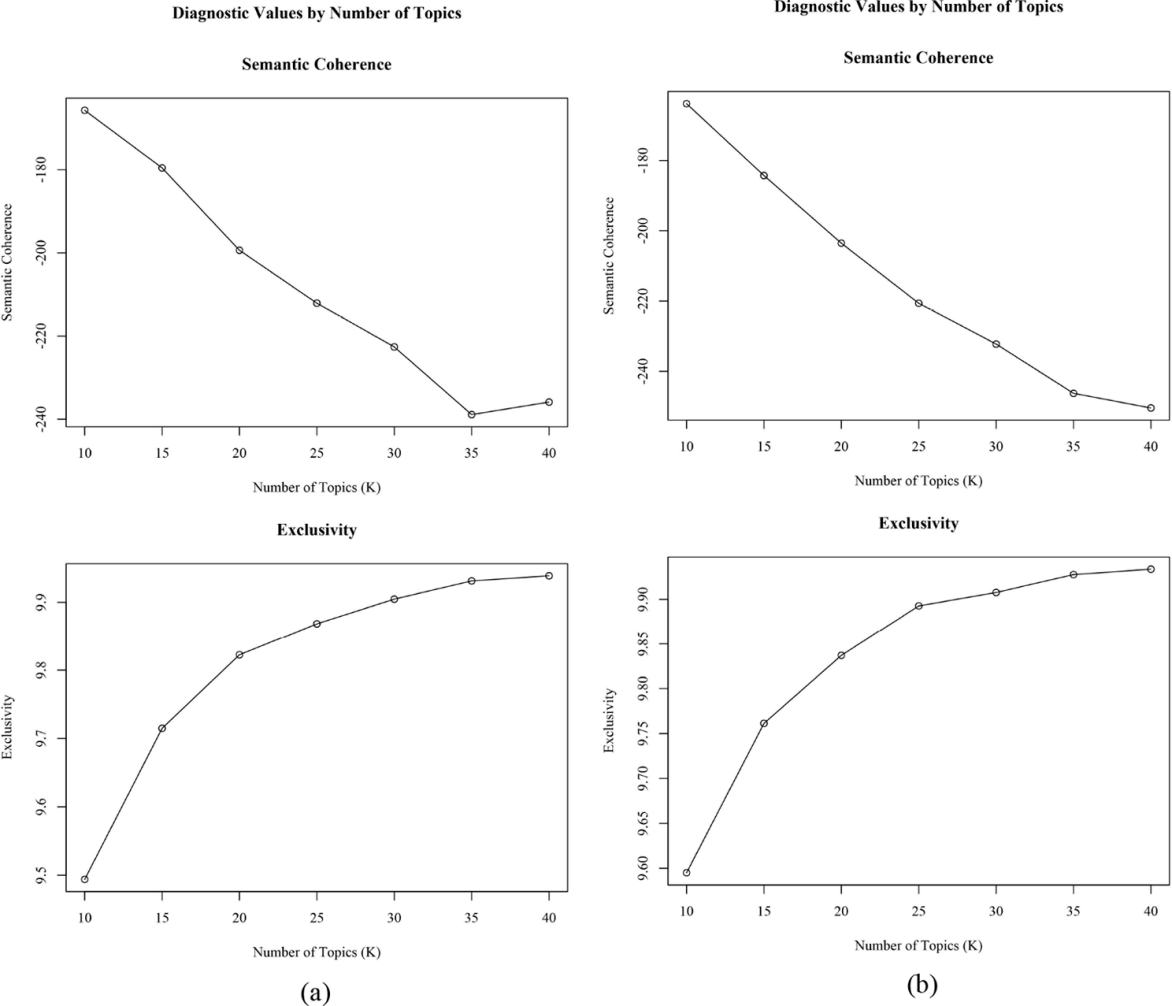
**a-b** Criteria of the selection of proper topic numbers in UK dataset (**a**) and India dataset (**b**)

It is crucial to determine the number of predicted topics. The package offers four evaluation criteria: held-out likelihood, semantic coherence, exclusivity, and model residuals. Particular attention was given to semantic coherence and exclusivity as they have been proven more valuable for the evaluation and judgement on the number of high-quality topics [38, 39]. A higher coherence indicates that multiple documents may contain similar top words from a topic, improving the interpretability of the topics [38]. A well-performed exclusivity means that high-probability words within a topic are less commonly present in other topics, thus representing the higher quality of the topic [40]. We tested a range of topic numbers from 10 to 40, hypothesizing that topics less than 10 would be insufficient for interpretation while topics more than 40 would be excessive. In this range, we also selected numbers that are multiples of 5. Figure 2 depicts the performance of the four criteria in both UK dataset (a) and India dataset (b) after iterations. Interestingly, semantic coherence exhibited a consistent decline in both countries while exclusivity climbed with the increase in topic numbers. Through manual examination, 25 seemed to be a proper number for both datasets, as semantic coherence stayed at a relatively balanced point and exclusivity reached a plateau. With the proper topic number determined, we performed topic model estimation (RQ1) and assessed the effect of metadata (RQ2).

#### Sentiment analysis and emotion categorization

The R package *#syuzhet* [41] was adopted in this study because of its capability of extracting emotions and sentiment-derived plot arcs from text using a variety of sentiment dictionaries, making it a versatile tool for analyzing the emotional context of social media posts [42, 43]. Two of the dictionaries, ‘NRC’ and ‘AFINN’, were applied to this study. ‘NRC’ contained a list of vocabulary and associations reflecting eight emotions (fear, anger, trust, sadness, disgust, anticipation, surprise, joy) and two moods (positive, negative), evoked by various elements within documents. It was utilized in this study to provide a more nuanced understanding of the overall sentiment in tweets. On the other hand, ‘AFINN’ was a list of pre-computed sentiment scores for English words, providing scales (from − 5 to + 5) to measure the intensity of sentiment. Given the brevity of tweets, this dictionary can help capture the intensity of sentiment in specific topics.

## Results and discussions

### Differences in topics between the UK and India

Specific topics are generated through STM estimation, and then manually labelled and categorized into different themes. Tables 2 and 3 have listed 25 topics and 5 themes in the UK and India, respectively. British people mainly focus on personal experiences (32.17%), government responses (23.02%), community support and volunteering (12.30%), impact on specific groups (11.18%), healthcare and safety measures (25.2%). In comparison, Indian people’s discussions on social media include public health and safety (24.74%), government responses (18.31%), social media and news (11.71%), coping strategies (17.36%), cooperations (27.88). From our results, personal experiences during lockdown are considered the top priority among British people whereas cooperations during the pandemic are a main focus of people based in India. The differences in topics and themes reveal two opposing cultural dimensions in the two countries: individualism in the UK and collectivism in India. This is in line with previous works such as [44] and [45]. More details will be discussed in RQ3.

**Table 2:** Topics among tweets in UK dataset

Themes and topics	Top 10 words
***Theme 1: Personal Experiences (32.17%)***	
Daily Issues	day, plea, friend, weekend, always, coronavirus, mention, coronavirus-outbreak, bank, rate
Home Life	house, UK-lockdown, gym, fit, flower, sun, clean, TikTok, train, bird
Family Plan	return, flight, war, situation, apart, easter-weekend, corona-UK, family, stream, live
Lifestyle	live, air, possible, travel, essential, fresh, beach, exercise, neighbour, run
Football	football, rainbow, wait, hour, talk, meme, player, reality, Chesham, cut
Appreciation	hero, patient, matter, huge, care, appreciate, frontline, cause, thank, staff
Personal Emotions	stupid, already, scare, profit, surprise, stop, pick, royal, channel, stress
Personal Life	birthday, today, bit, little, love, leather, sign, life, end, cake
***Theme 2: Government Responses (23.02%)***	
News and Information	BBC, inform, kingdom, Edinburgh, BBC-breakfast, news, face, blog, power, regard
Government Responses	coffee, prime, luck, prepare, minister, frequent, British, anxious, article, client
Public Actions	final, worse, infect, area, bad, Tesco, man, longer, waste, probably
Boris Johnson	Boris, Johnson, soon, fight, Borus, speedy, human, pull, Boris-Johnson, pray-for-Boris
National Responses	nation, forward, usual, newspaper, bread, bar, moment, advice, queen, second
Official Briefings	question, answer, brief, market, Chinese, account, press, fine, quiet, weather
***Theme 3: Community Support and Volunteering (12.30%)***	
Support	fund, charity, food, deliver, local, respond, support, domestic, team, busy
Community	fun, week, Covid, share, isolate, community, video, shop, continue, update
Volunteering	app, sooner, furlough, help, certain, kind, skill, base, volunteer, need
***Theme 4: Impact on Specific Groups (11.18%)***	
Job Concerns	thing, ill, job, really, disgrace, worry, risk, away, work, self-isolation
Vulnerable Groups	kid, India, park, Brexit, vulnerable, parent, miss, bear, rest, woman
Students	student, normal, poem, smile, artwork, mood, window, funny, approach, high
***Theme 5: Healthcare and Safety Measures (21.33%)***	
Lockdown	stay-safe, stay, safe, indoor, party, covid, open, nice, stay-at-home, laugh
Social Distancing	distance, social, order, eye, late, worst, lose, practice, ventil, dental
Hospital	hospital, coronavirus, London, covid, covid-UK, use, lockdown-UK, episode, clear, music
Confirmed Cases	confirm, case, test, Germany, April, York, Nigeria, increase, Spain, vaccine
Healthcare	key, worker, mental, health, line, doctor, NHS-hero, homeschool, story, wellbeing

**Table 3:** Topics among tweets in India dataset

Themes and topics	Top 10 words
***Theme 1: Public Health and Safety (24.74%)***	
Confirmed Cases	case, total, death, report, new, number, cross, far, confirm, rise
Safety Measures	meditation, start, door, immune, Noida, inspire, Tuesday-morning, design, way, ventil
Public Health	water, hand, air, woman, public, protect, import, session, station, impact
Anxiety	difficult, long, fear, thread, kid, time, away, Hydroxychloroquine, continue, battle
Urgent Situation	people, any, April, face, kill, village, dead, bank, religion, health
Pandemic Issues	coronavirus, corona, covid, virus, coronavirus-updates, lockdown-effect, global, man, Chinese-virus, corona-alert
***Theme 2: Government Responses (18.31%)***	
Social Actions	hospital, private, bed, cost, group, government, medical, age, step, police
Government Responses	up-police, study, locked-down, Arvind-Kejriwal, acquire, bible, big, treatment, differ, close
Relief Measures	minister, small, chief, PM-care, PM-relief-fund, corona-lockdown, digital, link, collect, month
Frontline Workers	worker, migrant, selfless, corona-warrior, tireless, live, day, quarantine, bit, love
National Responses	Coronavirus-India, Ashok-Gehlot, Bangalore, Indian, present, spray, citizen, social-distancing, open, crore
***Theme 3: Social Media and News (11.71%)***	
Social Media	social, Sakal, Sakal-media, Sakal-now, Sakal-news, distancing, news, viral-news, media, spread
Public Figures	Amit-Shah, Akshay-Kumar, rational, Narender-Modi, Rajnath-Singh, red-hill, distribute, kit, Mlkhattar, covid
City-Level News	test, covid, Indore-News, Vadodara, breaking-news, Ahmedabad, flower, Rajkot, Gandhinagar, trend
***Theme 4: Coping Strategies (17.36%)***	
Bathinda Quarantine	safe, lake, home, Bathinda-news, Bathinda-updates, Bathinda, comic, strip, inform, extend
Social Distancing	lockdown-extension, coronavirus-truth, song, covid-India, coffee, pharmacist-recruit, pharmacist, dalgona, guy, war
Fitness	day-fix, channel, workout-motive, workout, fitness-moto, gym-motive, social-distancing, fitness-motive, Ramayan, covid
Yoga	yoga-inspire, yoga-instructor, yoga-teach, city, yoga-stretch, family, affect, disease, friend, volunteer
***Theme 5: Cooperations (27.88%)***	
Stay Home Campaigns	stay-aware-stay-safe, India-fights-coronavirus, stay-home-safely, stay-home-India, lockdown-now, fight-against-corona, film, stay-home-stay-safe, real, period
Essentials	essentials, list, view, busy, clear, plea, attach, product, goods, request
National Unity	light, unity, fight, unit, switch, nation, together, tonight, let, power
Appreciation	help, human, situation, thank, best, hope, very, respect, always, soon
Global Efforts	world, coronavirus, countries, great, China, infect, need, action, effort, leader
Fundraising Support	support, fund, donate, care, sir, team, money, dear, relief, proud
Aid	amp, dure, video, food, place, Tablighi-jamaat, poor, member, outbreak, road

### Differences in the topics between popular and unpopular tweets in each country

**Fig. 3:**
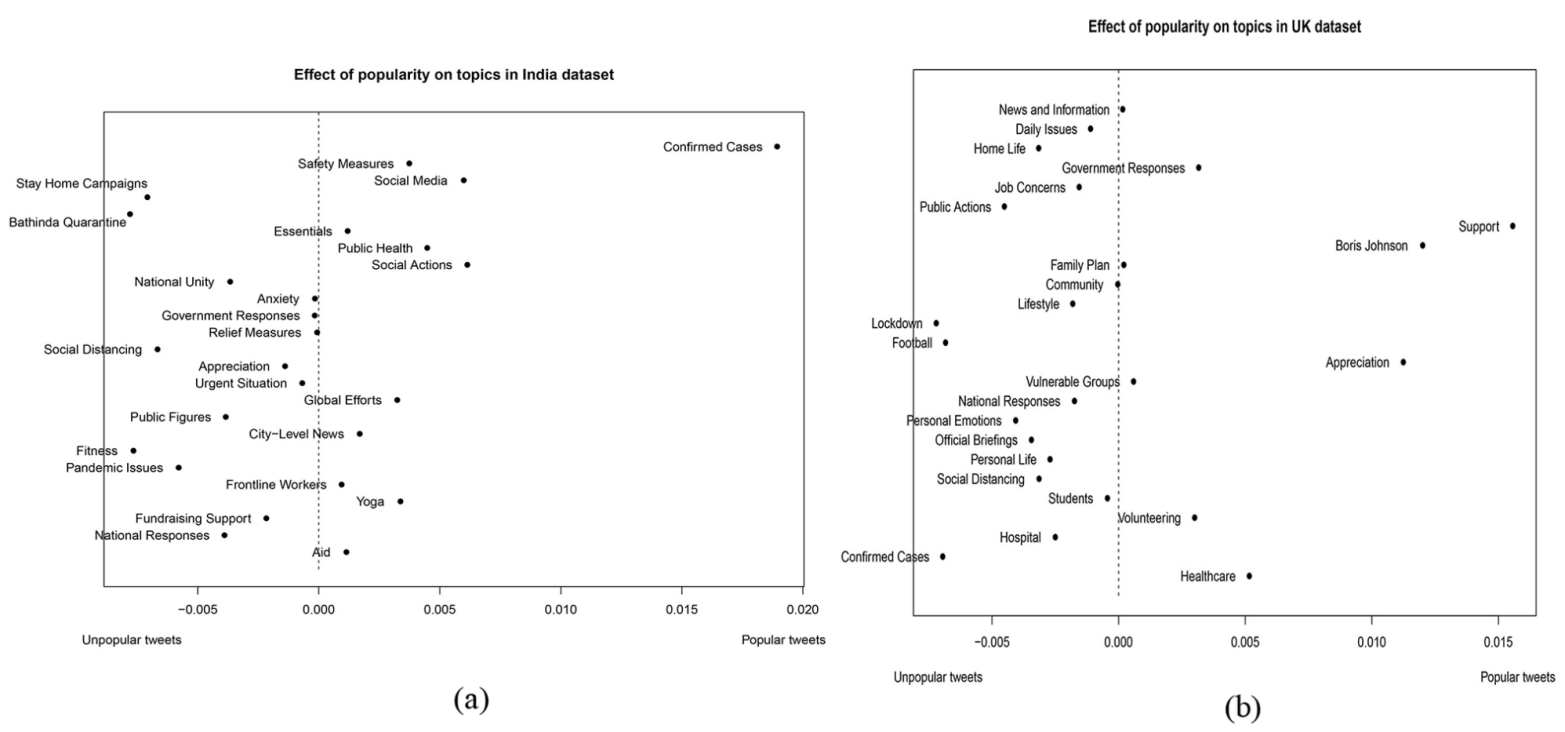
**a-b** Different topics from popular and unpopular tweets in UK dataset and India dataset

Figure 3 shows how popularity of tweets can affect topic distribution in the UK (a) and India (b). Topics located on the left side of the dotted line are those identified from unpopular tweets, while topics on the right are those identified from popular tweets. The farther the topics are from the dotted line, the more likely the topics are focused by popular / unpopular tweets. Obviously, popular tweets published in the UK have paid significant attention to support, Boris Johnson and expression of appreciation at the start of lockdown. In the UK, the concentration on Boris Johnson might be attributed to the political climate at that time. As Prime Minister, he was a central figure in the UK’s response to the pandemic, and his own contraction of the virus slightly before the beginning of lockdown likely led to increased discussion about him on Twitter [46]. Besides, the emphasis on support and appreciation could reflect the public’s response to the government’s measures, such as the efforts of healthcare workers and other essential practitioners during the lockdown [47].

In contrast, in India, the focus of popular tweets on confirmed cases of Covid-19 could be due to the rapidly increasing number of Covid-19 cases in this country during that period. The high number of cases likely leads to increased public concern and discussion about the pandemic’s progression. The Indian government’s measures, such as the nationwide lockdown announced in late March 2020, could have also contributed to the focus on confirmed cases as people sought updates and information about the pandemic [48]. Due to the existence of misinformation with the increase in public discussions on this issue, popular tweets, such as those published by official bloggers, play a crucial role in disseminating accurate, timely, and reliable information about Covid-19 [49].

Issues regarding lockdown have the highest probability to be heatedly discussed among unpopular tweets, including stay-home campaigns, Bathinda quarantine, social distancing in India and lockdown in the UK (topics located on the far left side of both figures). The impact on diverse groups of people has led to a variety of discussions among individual bloggers. For example, in the UK, lockdown brings specific challenges for new and expectant parents due to changes in perinatal services [50]. Lockdown also causes a significant impact on unpaid carers of individuals with disabilities, with changes in routines causing behavioral problems for those they care for and exacerbating financial struggles [51]. Besides, individual bloggers from India have posted their concerns about the situation of migrant construction-site workers, who faced unemployment, monetary issues, and social concerns such as discrimination, mistreatment, and lack of social assistance [52]. The class struggle intrinsic to Indian society is also highlighted [53]. In summary, individual bloggers have been proved to play a role in promoting health behaviors and coping strategies, as well as raising awareness about the pandemic’s impact on mental health and quality of life [54].

### Differences in people’s attitudes to Covid-19 between the UK and India

We start our sentiment analysis by simply categorizing all lexicons into positive, negative and neutral ones to examine sentiment distributions in both countries, in terms of the sentiment dictionary. A striking difference is that positive attitudes accounts for the most in the UK (40.90%) while much less in India (24.97%). Tweets posted in India are greatly dominated by neutral voices (58.38%). On the other hand, negative attitudes take up the smallest proportions in both the UK (22.35%) and India (16.65%). However, a simple sentiment categorization cannot provide insights into fine-grained investigation on the difference in people’s attitudes, thus we extracted emotions by means of the ‘NRC’ sentiment dictionary.

**Fig. 4:**
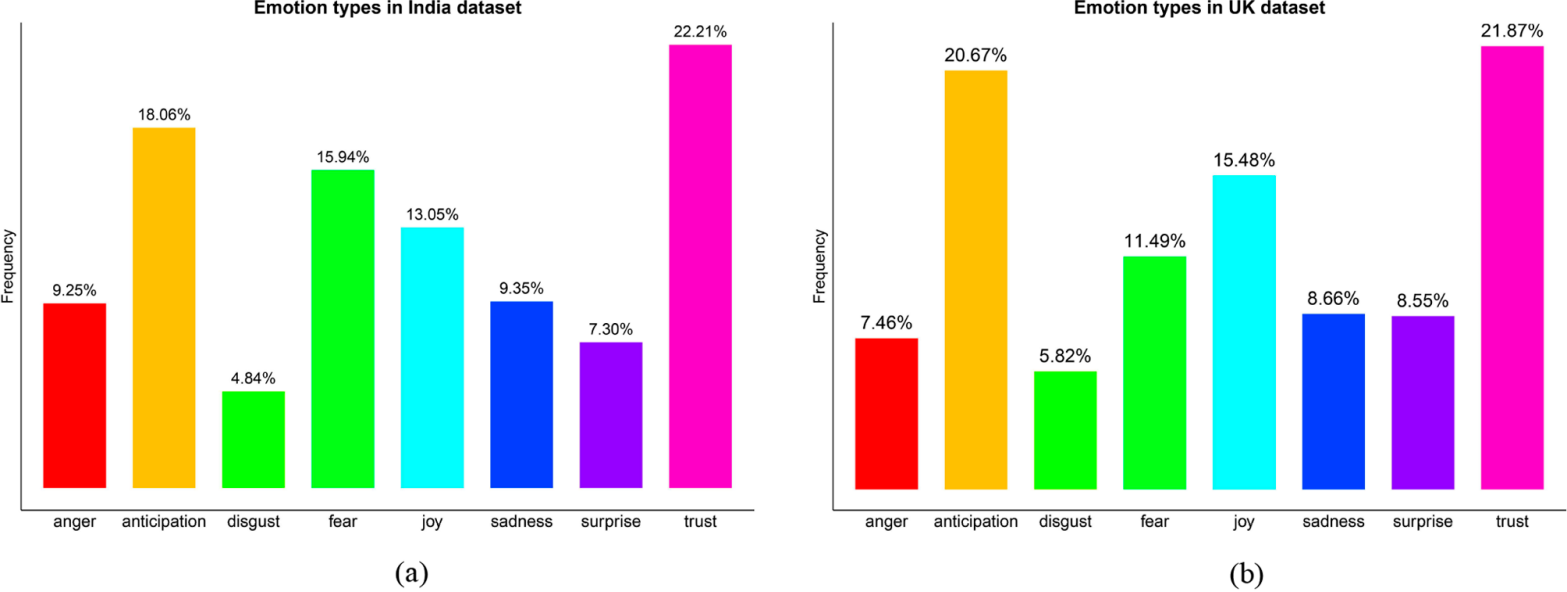
**a-b** The distribution of people’s emotions in UK dataset and India dataset

Figure 4 shows the distribution of people’s emotions in the UK (a) and India (b). Eight types of emotions are displayed on x-axis with their relevant proportions on y-axis. Obviously, in both countries, trust is the most prevalent emotion, with 21.87% in the UK and 22.21% in India. This is followed by anticipation, with 20.67% in the UK and 18.06% in India. Besides, both datasets show the lowest percentages of disgust among all types of emotions. Nevertheless, compared with the UK, tweets published in India contain more anger, fear and sadness (negative), whereas less joy and surprise (positive). This can further account for the significantly lower proportion of positive attitudes in India, as mentioned above.

To take a step further, this study performed sentiment analysis on specific topics to examine people’s attitudes towards difference aspects regarding Covid-19 right after lockdown announcement, especially those where sentiment is not explicitly expressed. To achieve this, the ‘AFINN’ sentiment dictionary was used here to calculate sentiment scores in each topic based on the most representative lexicons. We subsequently calculated the weighted average sentiment score for each theme, as different topics contain different numbers of lexicons, in terms of scores of each topic. Finally, we determined sentiment direction for each topic according to the sentiment score (*ps* for positive, *ng* for negative, *nt* for neutral), while we kept the scores for each theme as they can offer intensity of sentiment for further comparison.

Tables 4 and 5 have listed sentiment scores of specific topics and themes in the UK and India, respectively. In general, British people show relatively positive attitudes to community support and volunteering (2.59), followed by personal experiences (2.19), while Indian people feel positive about cooperations (3.51), government responses (2.26) and coping strategies (1.26). One noticeable finding is, in both countries, the sentiment towards the government’s response was different. Compared with the UK (0.03), the sentiment was generally more positive in India (2.26). In line with previous studies [13, 55], It has also been proved that the majority of Indian people are highly in favor of lockdown as a protective measure announced by Indian government.

**Table 4:** Sentiment scores in specific topics in UK dataset

Themes and topics	Sentiment scores
***Theme 1: Personal Experiences (2.19)***	
Daily Issues	ps
Home Life	nt
Family Plan	ps
Lifestyle	ps
Football	nt
Appreciation	ps
Personal Emotions	ps
Personal Life	nt
***Theme 2: Government Responses (0.03)***	
News and Information	ps
Government Responses	ps
Public Actions	ng
Boris Johnson	ps
National Responses	ng
Official Briefings	ng
***Theme 3: Community Support and Volunteering (2.59)***	
Support	ps
Community	ps
Volunteering	ps
***Theme 4: Impact on Specific Groups (-0.27)***	
Job Concerns	ng
Vulnerable Groups	ng
Students	ps
***Theme 5: Healthcare and Safety Measures (-0.16)***	
Lockdown	nt
Social Distancing	nt
Hospital	ps
Confirmed Cases	ng
Healthcare	nt

**Table 5:** Sentiment scores in specific topics in India dataset

Themes and topics	Sentiment scores
***Theme 1: Public Health and Safety (-0.79)***	
Confirmed Cases	ng
Safety Measures	ps
Public Health	ps
Anxiety	ng
Urgent Situation	ng
Pandemic Issues	ps
***Theme 2: Government Responses (2.26)***	
Social Actions	ps
Government Responses	ps
Relief Measures	ng
Frontline Workers	ps
National Responses	nt
***Theme 3: Social Media and News (-0.96)***	
Social Media	nt
Public Figures	nt
City-Level News	ps
***Theme 4: Coping Strategies (1.26)***	
Bathinda Quarantine	ps
Social Distancing	ps
Fitness	ng
Yoga	ps
***Theme 5: Cooperations (3.51)***	
Stay Home Campaigns	ps
Essentials	nt
National Unity	ps
Appreciation	ps
Global Efforts	nt
Fundraising Support	ps
Aid	ps

This can be attributed to several reasons. Firstly, many Indian citizens believe that lockdown policies are necessary to curb the spread of COVID-19 [56]. has found that more than a half of participants agree that lockdown is essential to control the outbreak of the virus. However, this belief can conversely be influenced by the effectiveness of lockdown in slowing down the peak of the pandemic, as indicated in [57]. Secondly, the Indian public’s support for lockdown measures seems to be affected by their instrumental attitudes, which are based on the outcomes of the lockdown, rather than their experiential attitudes, which are based on their experiences [58]. This suggests that many Indian people support lockdown because they believe these measures will lead to positive outcomes, such as reducing the spread of the virus, rather than because they find the experience of being pleasant during lockdown periods.

Combining the results here with those in RQ1 above, generally, British people pay much attention to and feel greatly positive about their personal experiences at the start of lockdown, while Indian people think highly of cooperations, revealing the individualism-collectivism cultural dimension. This dimension has been found to significantly impact the effectiveness of pandemic controls [59, 60]. has demonstrated greater compliance and adherence to social norms in collectivist cultures, as people highly emphasize their civic responsibility [61]. For instance, a higher degree of collectivist culture can be associated with greater probability of wearing masks and more trust medical practitioners [62]. However, in spite of that, lockdown experiences a failure to curb the growth of conformed cases in India, compared with the UK [63]. One possible reason for this is the shorter social distance between people. Lockdown has brought India severe starvation [64], forcing Indian people to go outside to purchase daily essentials [63], which reduces the effect of social distancing. This, alongside with other lockdown violations, indicates a lack of effective community mobilization [63].

In general, while cultural preferences for individualism or collectivism do play a role in shaping the response to Covid-19, they are part of a broader set of factors that determine the success of lockdowns and pandemic management. As [65] has found, the benefits of collectivism do not imply its superiority over individualism in other circumstances. With the increasing need of vaccination and effective governance in halting the spread of Covid-19, underdeveloped countries characterized by collectivism may find themselves at a greater disadvantage [65]. To solve this, the relationship between cultural orientation and pandemic situation can be mediated by other factors such as public responses to government policies [59].

## Conclusions

RQ1 focuses on the difference in the topics on Covid-19 between the UK and India at the start of lockdown. Based on our results, personal experiences during lockdown are considered the top priority among British people whereas they participate the least in discussions about how specific groups can be affected by Covid-19. In comparison, people based in India have placed the most focus on cooperations during the pandemic while the least on social media and news reports. Besides, safety measures, government responses and cooperative supports are common themes in both countries.

RQ2 distinguishes the difference in the topics on Covid-19 between popular and unpopular tweets in each country at the start of lockdown. Popular tweets published in the UK have paid significant attention to supports, Boris Johnson and expression of appreciation, while confirmed cases of Covid-19 have been placed much emphasis among popular tweets in India. Moreover, issues regarding lockdown have the highest probability to be heatedly discussed among unpopular tweets, including stay-home campaigns, Bathinda quarantine, social distancing in India and lockdown in the UK.

RQ3 revolves around the difference in people’s attitudes toward COVID-19 between the UK and India at the start of lockdown. In general, positive attitudes accounts for the most in the UK while much less in India. Tweets posted in India are greatly dominated by neutral voices. Negative attitudes take up the smallest proportions in both countries. According to emotion extraction, trust and anticipation are the most prevalent emotions in both countries, while India has experienced more negative emotions (anger, fear and sadness) and less positive emotions (joy and surprise) than the UK. For specific themes, British people feel positive about community support and volunteering, personal experiences and government responses, while Indian people feel positive about cooperations, government responses and coping strategies. Public health situations raise negative sentiment both in the UK and India.

This study undoubtedly provides implications for crisis communication strategies and public health policy making. For example, the underlying cultural values come to the forefront and influence how societies from different cultures respond. Individualistic societies, such as the UK, may lead to a greater emphasis on personal freedom and autonomy, focusing more on individual responsibilities. Thus, governments need to balance individual liberty with public health measures in these societies. On the other hand, collectivistic societies, such as India, often show a greater concern for the impact on the community and the importance of cooperative actions, highlighting the well-being of the community and social solidarity. Therefore, policies could leverage community networks to enhance public health compliance.

The identification of topics between popular and unpopular tweets may reflect and reshape public discussions. Heatedly discussed topics, such as Boris Johnson in the UK and confirmed cases in India, can be challenged and misperceived due to the existence of fake news, as these topics are of high interest. Hence, official and famous bloggers are anticipated to strictly confirm the authenticity of information. On the other hand, the focus on lockdown issues by individual bloggers in both countries underscores the role of social media influencers in shaping public discourse, filling information gaps and address specific concerns that may not be covered by popular tweets. In sum, understanding public concerns and the type of content that gains popularity on social media can help authorities and health organizations to better manage public expectations and tailor their crisis communication to improve public engagement and compliance with health measures.

This study is not free of limitations. Subjectivity lies in the process of selecting proper topic numbers and naming different topics and themes. Further research is needed to adopt more complicated algorithms to validate the feasibility of alternative topic numbers and to better cluster lexicons into latent topics, thus more rationalizing the interpretation of each topic and theme. Future works can also continue to expand along with the assessment of effects of tweets popularity, including but not limited to the selection of more reasonable criteria on distinguishing popular tweets from unpopular ones.

## Data Availability

The datasets used and analyzed during the current study are available upon request.
